# 2-Bromo-1-(1-phenyl­sulfonyl-1*H*-indol-3-yl)propan-1-one

**DOI:** 10.1107/S1600536814002864

**Published:** 2014-02-15

**Authors:** C. Ramathilagam, P. R. Umarani, V. Saravanan, A. K. Mohanakrishnan, B. Gunasekaran, V. Manivannan

**Affiliations:** a Department of Physics, AMET University, Kanathur, Chennai 603 112, India; b Department of Physics, Kundavai Nachiyar Govt College for Women, Thanjavur 613 007, India; c Department of Organic Chemistry, University of Madras, Guindy Campus, Chennai 600 025, India; d Department of Physics & Nano Technology, SRM University, SRM Nagar, Kattankulathur, Kancheepuram Dist, Chennai 603 203 Tamil Nadu, India; e Department of Research and Development, PRIST University, Vallam, Thanjavur 613 403, Tamil Nadu, India

## Abstract

In the title compound, C_17_H_14_BrNO_3_S, the phenyl ring makes a dihedral angle of 89.78 (16)° with the plane of the indole ring system. The terminal Br atom and the methyl group are disordered over two sets of sites, with site occupancies of 0.860 (2) and 0.140 (2). In the crystal, mol­ecules are linked into a chain along the *b-*axis direction by weak C—H⋯O hydrogen bonds. The chains are further linked by C—H⋯π inter­actions, forming layers parallel to the *bc* plane.

## Related literature   

For the biological activity of indole derivatives, see: Andreani *et al.* (2001 ▶); Singh *et al.* (2000 ▶); Pomarnacka & Kozlarska-Kedra (2003 ▶); Srivastava & Pandeya (2011 ▶). For related structures, see: Umadevi *et al.* (2013 ▶); Kanchanadevi *et al.* (2014 ▶).
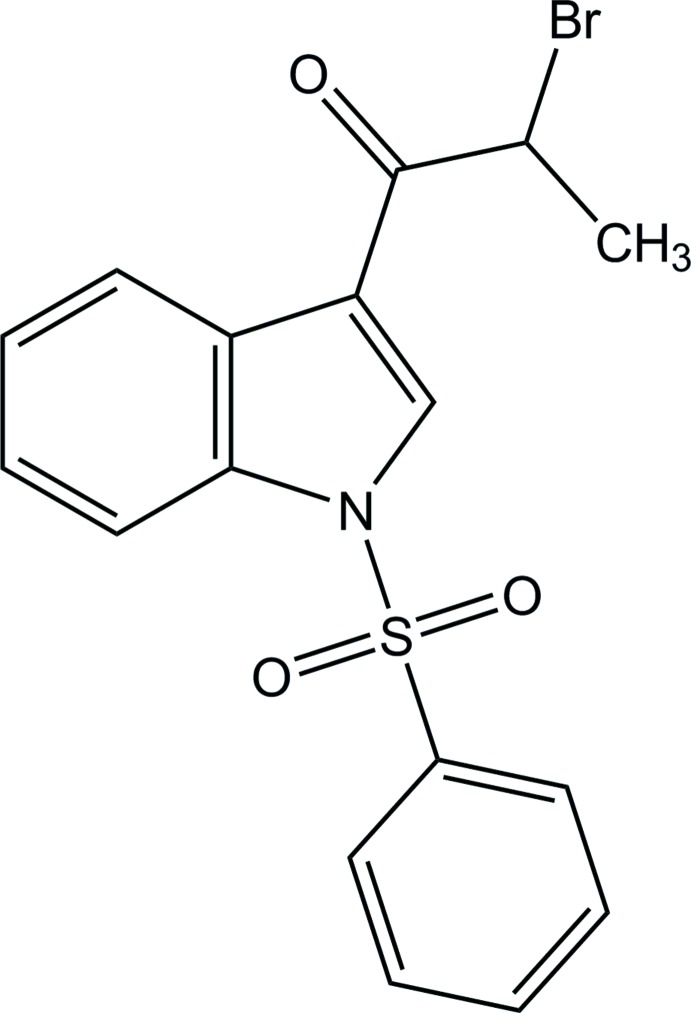


## Experimental   

### 

#### Crystal data  

C_17_H_14_BrNO_3_S
*M* *_r_* = 392.26
Monoclinic, 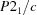

*a* = 8.7539 (3) Å
*b* = 10.9968 (4) Å
*c* = 17.5801 (7) Å
β = 99.231 (2)°
*V* = 1670.43 (11) Å^3^
*Z* = 4
Mo *K*α radiation
μ = 2.60 mm^−1^
*T* = 295 K
0.35 × 0.25 × 0.25 mm

#### Data collection  

Bruker APEXII CCD diffractometer
Absorption correction: multi-scan (*SADABS*; Sheldrick, 1996 ▶) *T*_min_ = 0.416, *T*_max_ = 0.522
15009 measured reflections
4141 independent reflections
2334 reflections with *I* > 2σ(*I*)
*R*_int_ = 0.034

#### Refinement  

*R*[*F* ^2^ > 2σ(*F* ^2^)] = 0.050
*wR*(*F* ^2^) = 0.149
*S* = 1.03
4141 reflections
222 parameters
5 restraints
H-atom parameters constrained
Δρ_max_ = 0.41 e Å^−3^
Δρ_min_ = −0.51 e Å^−3^

### 

Data collection: *APEX2* (Bruker, 2008 ▶); cell refinement: *SAINT* (Bruker, 2008 ▶); data reduction: *SAINT* (Bruker, 2008 ▶); program(s) used to solve structure: *SHELXS97* (Sheldrick, 2008 ▶); program(s) used to refine structure: *SHELXL97* (Sheldrick, 2008 ▶); molecular graphics: *PLATON* (Spek, 2009 ▶); software used to prepare material for publication: *SHELXL97* (Sheldrick, 2008 ▶).

## Supplementary Material

Crystal structure: contains datablock(s) I. DOI: 10.1107/S1600536814002864/is5339sup1.cif

Structure factors: contains datablock(s) I. DOI: 10.1107/S1600536814002864/is5339Isup2.hkl

Supporting information file. DOI: 10.1107/S1600536814002864/is5339Isup3.cml

CCDC reference: 985849

Additional supporting information:  crystallographic information; 3D view; checkCIF report

## Figures and Tables

**Table 1:** Hydrogen-bond geometry (Å, °) *Cg*2 is the centroid of the C1–C6 ring.

*D*—H⋯*A*	*D*—H	H⋯*A*	*D*⋯*A*	*D*—H⋯*A*
C8—H8⋯O1^i^	0.93	2.57	3.312 (4)	137
C10—H10⋯O1^i^	0.93	2.44	3.280 (4)	150
C12—H12⋯*Cg*2^ii^	0.93	2.72	3.528 (2)	146

Symmetry codes: (i) 
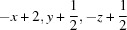
; (ii) 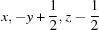.

**Fig. 1.**
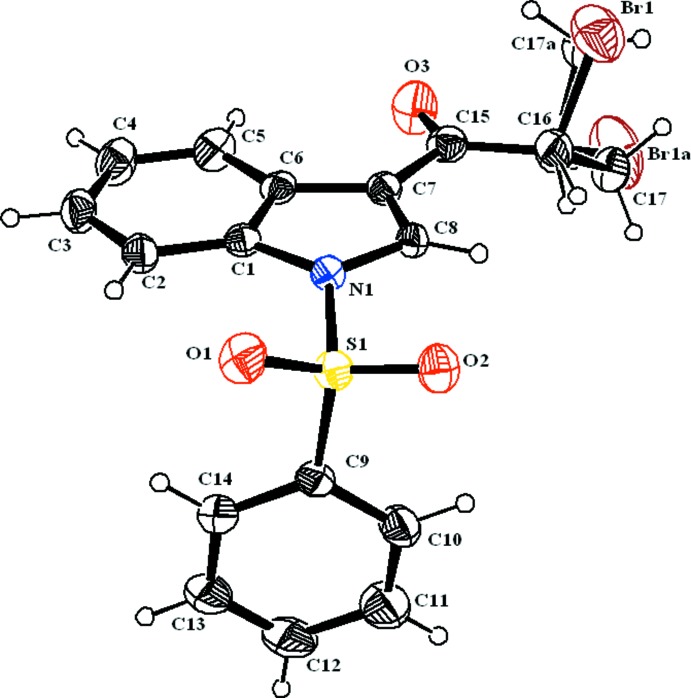
The molecular structure of the title compound, with atom labels and 30% probability displacement ellipsoids for non-H atoms.

**Fig. 2.**
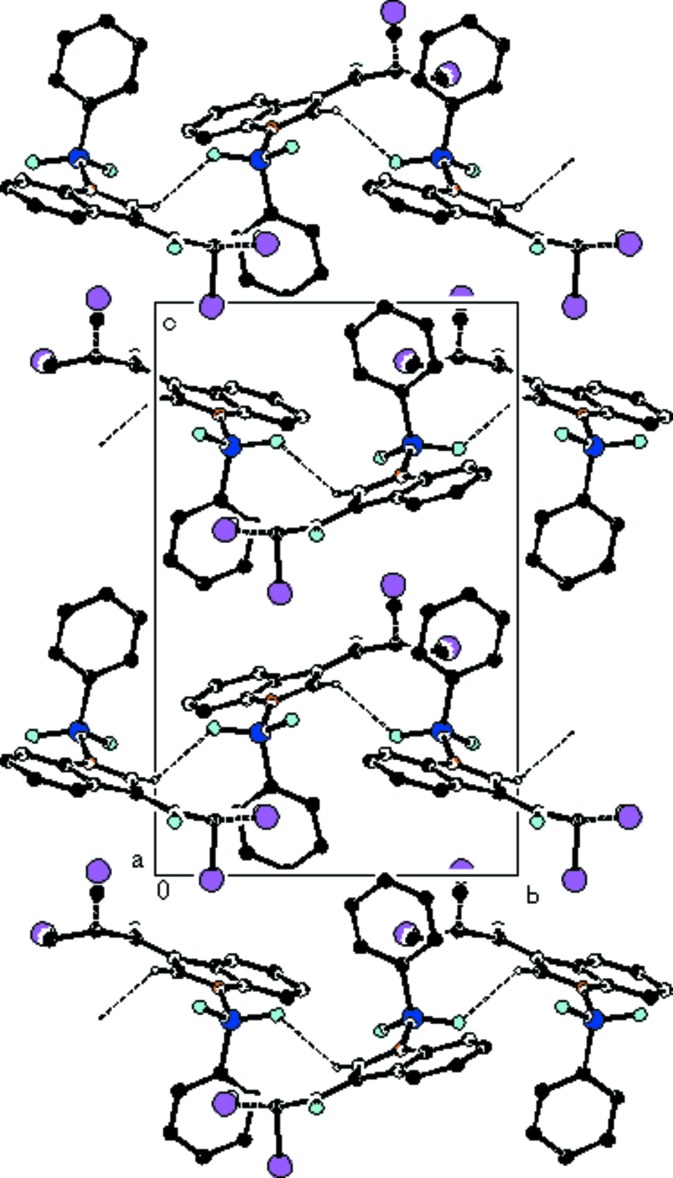
The packing diagram of the title compound, viewed down the a axis. Hydrogen bonds are shown as dashed lines.

## supplementary crystallographic information

### 1. Comment

Indole derivatives exhibit antibacterial, antifungal (Singh *et al.*, 2000) and antitumour activities (Andreani *et al.*, 2001). These derivatives also exhibit antimicrobial, antibiotic, analgesic, anticancer and anti-HIV (Pomarnacka & Kozlarska-Kedra, 2003; Srivastava & Pandeya, 2011) activities.

The geometric parameters of the title molecule (Fig. 1) agree well with reported similar structures (Umadevi *et al.*, 2013; Kanchanadevi *et al.*, 2014). The phenyl ring makes a dihedral angle of 89.78 (16)° with the indole ring system. The terminal bromine atom and the methyl group are disordered over two positions with site occupancies of 0.860 (2) and 0.140 (2). The sum of bond angles around the atom N1 [356.7 (3) °] indicates *sp^2^* hybridized state of atom N1 in the molecule. The crystal packing is controlled by weak C—H···O and C—H···π interactions (Table 1).

### 2. Experimental

A solution of 1-[1-(phenylsulfonyl)-1*H*-indol-3-yl]propan-1-one (1 g, 3.194 mmol) and PTT (phenyltrimethylammonium tribromide) (1.32 g, 3.514 mmol) in dry THF (20 ml) was stirred at room temperature for 3 h. After completion of the reaction (monitored by TLC), it was poured into crushed ice (100 g). The solid obtained was filtered and washed with MeOH (5 ml) to afford 2-bromo-1-[1-(phenylsulfonyl)-1*H*-indol-3-yl]propan-1-one (1.14 g, yield 91%; melting point 130–132 °C).

### 3. Refinement

The terminal bromine atom and the methyl group are disordered over two positions. The site occupancy factors of disordered atoms were refined to 0.860 (2) and 0.140 (2). In the refinement, *EADP* was used for atoms C17 and C17A. The bond distances of C16—C17 and C16—C17A were restrained to be 1.5200 (1) and 1.5200 (5) Å, respectively, and the distances of C16—Br1 and C16—Br1A were restrained to be 1.9100 (1) and 1.9100 (5) Å, respectively. Also the distance of C17A···Br1A was restrained to be 2.85 (1) Å. H atoms were positioned geometrically and refined using riding model, with C—H = 0.93 Å and *U*_iso_(H) = 1.2*U*_eq_(C) for aromatic C—H, C—H = 0.98 Å and *U*_iso_(H) = 1.2*U*_eq_(C) for C—H, C—H = 0.96 Å and *U*_iso_(H) = 1.5*U*_eq_(C) for CH_3_.

### Crystal data

**Table:**

C_17_H_14_BrNO_3_S	*F*(000) = 792
*M**_r_* = 392.26	*D*_x_ = 1.560 Mg m^−^^3^
Monoclinic, *P*2_1_/*c*	Mo *K*α radiation, λ = 0.71073 Å
Hall symbol: -P 2ybc	Cell parameters from 4350 reflections
*a* = 8.7539 (3) Å	θ = 2.2–28.4°
*b* = 10.9968 (4) Å	µ = 2.60 mm^−^^1^
*c* = 17.5801 (7) Å	*T* = 295 K
β = 99.231 (2)°	Block, yellow
*V* = 1670.43 (11) Å^3^	0.35 × 0.25 × 0.25 mm
*Z* = 4	

### Data collection

**Table:**

Bruker APEXII CCD diffractometer	4141 independent reflections
Radiation source: fine-focus sealed tube	2334 reflections with *I* > 2σ(*I*)
Graphite monochromator	*R*_int_ = 0.034
Detector resolution: 0 pixels mm^-1^	θ_max_ = 28.5°, θ_min_ = 2.2°
ω and φ scans	*h* = −11→8
Absorption correction: multi-scan (*SADABS*; Sheldrick, 1996)	*k* = −10→14
*T*_min_ = 0.416, *T*_max_ = 0.522	*l* = −21→23
15009 measured reflections	

### Refinement

**Table:**

Refinement on *F*^2^	Secondary atom site location: difference Fourier map
Least-squares matrix: full	Hydrogen site location: inferred from neighbouring sites
*R*[*F*^2^ > 2σ(*F*^2^)] = 0.050	H-atom parameters constrained
*wR*(*F*^2^) = 0.149	*w* = 1/[σ^2^(*F*_o_^2^) + (0.0673*P*)^2^ + 0.7073*P*] where *P* = (*F*_o_^2^ + 2*F*_c_^2^)/3
*S* = 1.03	(Δ/σ)_max_ = 0.001
4141 reflections	Δρ_max_ = 0.41 e Å^−^^3^
222 parameters	Δρ_min_ = −0.51 e Å^−^^3^
5 restraints	Extinction correction: *SHELXL97* (Sheldrick, 2008), Fc^*^=kFc[1+0.001xFc^2^λ^3^/sin(2θ)]^-1/4^
Primary atom site location: structure-invariant direct methods	Extinction coefficient: 0.0037 (9)

### Fractional atomic coordinates and isotropic or equivalent isotropic displacement parameters (Å^2^)

**Table:**

	*x*	*y*	*z*	*U*_iso_*/*U*_eq_	Occ. (<1)
C1	0.7170 (3)	0.2542 (3)	0.30787 (16)	0.0382 (7)	
C2	0.6804 (4)	0.1348 (3)	0.28691 (18)	0.0480 (8)	
H2	0.7480	0.0850	0.2655	0.058*	
C3	0.5365 (4)	0.0944 (3)	0.2998 (2)	0.0558 (9)	
H3	0.5071	0.0148	0.2870	0.067*	
C4	0.4359 (4)	0.1688 (3)	0.3310 (2)	0.0627 (10)	
H4	0.3398	0.1387	0.3380	0.075*	
C5	0.4753 (4)	0.2878 (3)	0.3522 (2)	0.0552 (9)	
H5	0.4072	0.3373	0.3735	0.066*	
C6	0.6184 (3)	0.3308 (3)	0.34090 (16)	0.0392 (7)	
C7	0.6963 (3)	0.4468 (2)	0.35599 (16)	0.0391 (7)	
C8	0.8349 (3)	0.4370 (2)	0.33195 (16)	0.0375 (7)	
H8	0.9074	0.4992	0.3341	0.045*	
C9	0.8963 (3)	0.3192 (3)	0.15480 (17)	0.0427 (7)	
C10	0.9085 (4)	0.4343 (3)	0.12403 (19)	0.0587 (9)	
H10	0.9658	0.4947	0.1527	0.070*	
C11	0.8349 (5)	0.4573 (4)	0.0508 (2)	0.0803 (12)	
H11	0.8417	0.5339	0.0292	0.096*	
C12	0.7501 (5)	0.3665 (4)	0.0088 (2)	0.0820 (13)	
H12	0.6992	0.3830	−0.0407	0.098*	
C13	0.7405 (5)	0.2530 (4)	0.0393 (2)	0.0804 (13)	
H13	0.6845	0.1926	0.0101	0.096*	
C14	0.8121 (4)	0.2279 (3)	0.1120 (2)	0.0614 (9)	
H14	0.8050	0.1508	0.1329	0.074*	
C15	0.6362 (4)	0.5538 (3)	0.39046 (19)	0.0511 (8)	
C16	0.7409 (4)	0.6635 (2)	0.40341 (9)	0.0651 (10)	
H16A	0.8186	0.6547	0.3694	0.078*	0.860 (2)
H16B	0.7916	0.6656	0.3576	0.078*	0.140 (2)
Br1	0.84813 (7)	0.65595 (6)	0.50680 (4)	0.0973 (3)	0.860 (2)
C17	0.6681 (10)	0.7883 (4)	0.3880 (5)	0.0539 (15)	0.860 (2)
H17A	0.7036	0.8411	0.4307	0.081*	0.860 (2)
H17B	0.5575	0.7812	0.3817	0.081*	0.860 (2)
H17C	0.6973	0.8215	0.3419	0.081*	0.860 (2)
Br1A	0.6298 (11)	0.8071 (6)	0.3977 (7)	0.132 (4)	0.140 (2)
C17A	0.875 (2)	0.6591 (18)	0.4701 (12)	0.0539 (15)	0.140 (2)
H17D	0.9557	0.7124	0.4596	0.081*	0.140 (2)
H17E	0.9138	0.5775	0.4763	0.081*	0.140 (2)
H17F	0.8396	0.6846	0.5166	0.081*	0.140 (2)
N1	0.8539 (3)	0.3213 (2)	0.30363 (13)	0.0384 (6)	
O1	1.0168 (3)	0.16368 (19)	0.25653 (14)	0.0591 (6)	
O2	1.1079 (2)	0.3760 (2)	0.27067 (13)	0.0526 (6)	
O3	0.5058 (3)	0.5548 (2)	0.40600 (18)	0.0778 (8)	
S1	0.98775 (9)	0.29060 (7)	0.24862 (4)	0.0422 (2)	

### Atomic displacement parameters (Å^2^)

**Table:**

	*U*^11^	*U*^22^	*U*^33^	*U*^12^	*U*^13^	*U*^23^
C1	0.0438 (16)	0.0389 (15)	0.0298 (16)	0.0014 (13)	−0.0003 (12)	0.0057 (12)
C2	0.059 (2)	0.0392 (17)	0.0421 (19)	−0.0028 (14)	−0.0019 (15)	0.0011 (13)
C3	0.060 (2)	0.0464 (18)	0.056 (2)	−0.0136 (17)	−0.0088 (16)	0.0034 (16)
C4	0.0454 (19)	0.065 (2)	0.074 (3)	−0.0139 (18)	−0.0001 (17)	0.0101 (19)
C5	0.0441 (18)	0.058 (2)	0.063 (2)	0.0016 (16)	0.0069 (15)	0.0098 (17)
C6	0.0413 (16)	0.0405 (15)	0.0340 (16)	0.0009 (13)	0.0004 (12)	0.0080 (12)
C7	0.0454 (16)	0.0353 (15)	0.0355 (16)	0.0031 (13)	0.0034 (12)	0.0027 (12)
C8	0.0464 (17)	0.0298 (14)	0.0357 (16)	−0.0021 (12)	0.0047 (12)	−0.0002 (11)
C9	0.0470 (17)	0.0435 (17)	0.0393 (17)	0.0018 (13)	0.0115 (13)	−0.0037 (13)
C10	0.083 (2)	0.0438 (19)	0.047 (2)	−0.0007 (17)	0.0053 (18)	−0.0021 (15)
C11	0.118 (4)	0.063 (2)	0.056 (3)	0.003 (2)	0.004 (2)	0.010 (2)
C12	0.097 (3)	0.107 (4)	0.037 (2)	−0.008 (3)	−0.004 (2)	−0.001 (2)
C13	0.099 (3)	0.098 (3)	0.042 (2)	−0.033 (3)	0.008 (2)	−0.012 (2)
C14	0.078 (2)	0.061 (2)	0.047 (2)	−0.0192 (19)	0.0146 (18)	−0.0042 (17)
C15	0.057 (2)	0.051 (2)	0.047 (2)	0.0062 (16)	0.0141 (16)	0.0018 (14)
C16	0.080 (3)	0.051 (2)	0.070 (3)	−0.0090 (18)	0.029 (2)	−0.0180 (17)
Br1	0.0956 (5)	0.0997 (5)	0.0870 (5)	−0.0029 (3)	−0.0142 (3)	−0.0100 (3)
C17	0.067 (4)	0.023 (2)	0.071 (4)	0.016 (2)	0.008 (3)	0.009 (2)
Br1A	0.118 (6)	0.078 (3)	0.192 (7)	0.027 (3)	0.004 (4)	−0.038 (4)
C17A	0.067 (4)	0.023 (2)	0.071 (4)	0.016 (2)	0.008 (3)	0.009 (2)
N1	0.0432 (14)	0.0363 (13)	0.0356 (14)	−0.0015 (10)	0.0062 (10)	−0.0010 (10)
O1	0.0659 (15)	0.0400 (12)	0.0727 (17)	0.0185 (10)	0.0151 (12)	0.0072 (10)
O2	0.0369 (11)	0.0555 (13)	0.0640 (15)	−0.0048 (10)	0.0040 (10)	−0.0037 (11)
O3	0.0651 (17)	0.0646 (16)	0.111 (2)	0.0071 (13)	0.0367 (16)	−0.0143 (15)
S1	0.0431 (4)	0.0378 (4)	0.0458 (5)	0.0067 (3)	0.0078 (3)	0.0012 (3)

### Geometric parameters (Å, º)

**Table:**

C1—C2	1.387 (4)	C12—C13	1.366 (6)
C1—C6	1.398 (4)	C12—H12	0.9300
C1—N1	1.420 (4)	C13—C14	1.359 (5)
C2—C3	1.388 (5)	C13—H13	0.9300
C2—H2	0.9300	C14—H14	0.9300
C3—C4	1.379 (5)	C15—O3	1.216 (4)
C3—H3	0.9300	C15—C16	1.511 (4)
C4—C5	1.389 (5)	C16—C17	1.5190 (10)
C4—H4	0.9300	C16—C17A	1.520 (5)
C5—C6	1.382 (4)	C16—Br1A	1.849 (4)
C5—H5	0.9300	C16—Br1	1.9092 (10)
C6—C7	1.450 (4)	C16—H16A	0.9800
C7—C8	1.352 (4)	C16—H16B	0.9800
C7—C15	1.460 (4)	C17—H17A	0.9600
C8—N1	1.386 (3)	C17—H17B	0.9600
C8—H8	0.9300	C17—H17C	0.9600
C9—C10	1.388 (4)	C17A—H17D	0.9600
C9—C14	1.392 (5)	C17A—H17E	0.9600
C9—S1	1.743 (3)	C17A—H17F	0.9600
C10—C11	1.368 (5)	N1—S1	1.669 (2)
C10—H10	0.9300	O1—S1	1.422 (2)
C11—C12	1.384 (6)	O2—S1	1.417 (2)
C11—H11	0.9300		
			
C2—C1—C6	123.3 (3)	C13—C14—C9	119.1 (3)
C2—C1—N1	129.7 (3)	C13—C14—H14	120.5
C6—C1—N1	107.0 (2)	C9—C14—H14	120.5
C1—C2—C3	115.9 (3)	O3—C15—C7	121.0 (3)
C1—C2—H2	122.0	O3—C15—C16	121.8 (3)
C3—C2—H2	122.0	C7—C15—C16	117.1 (3)
C4—C3—C2	121.9 (3)	C15—C16—C17	117.9 (4)
C4—C3—H3	119.0	C15—C16—C17A	117.7 (10)
C2—C3—H3	119.0	C15—C16—Br1A	111.9 (4)
C3—C4—C5	121.3 (3)	C17A—C16—Br1A	113.8 (6)
C3—C4—H4	119.4	C15—C16—Br1	107.61 (19)
C5—C4—H4	119.4	C17—C16—Br1	109.7 (4)
C6—C5—C4	118.4 (3)	C15—C16—H16A	107.0
C6—C5—H5	120.8	C17—C16—H16A	107.0
C4—C5—H5	120.8	Br1—C16—H16A	107.0
C5—C6—C1	119.2 (3)	C15—C16—H16B	103.8
C5—C6—C7	133.2 (3)	C17A—C16—H16B	103.8
C1—C6—C7	107.6 (2)	Br1A—C16—H16B	103.8
C8—C7—C6	107.0 (2)	C16—C17—H17A	109.5
C8—C7—C15	126.5 (3)	C16—C17—H17B	109.5
C6—C7—C15	126.5 (3)	H17A—C17—H17B	109.5
C7—C8—N1	110.6 (2)	C16—C17—H17C	109.5
C7—C8—H8	124.7	H17A—C17—H17C	109.5
N1—C8—H8	124.7	H17B—C17—H17C	109.5
C10—C9—C14	121.0 (3)	C16—C17A—H17D	109.5
C10—C9—S1	118.7 (2)	C16—C17A—H17E	109.5
C14—C9—S1	120.3 (2)	H17D—C17A—H17E	109.5
C11—C10—C9	118.8 (3)	C16—C17A—H17F	109.5
C11—C10—H10	120.6	H17D—C17A—H17F	109.5
C9—C10—H10	120.6	H17E—C17A—H17F	109.5
C10—C11—C12	120.0 (4)	C8—N1—C1	107.9 (2)
C10—C11—H11	120.0	C8—N1—S1	122.02 (19)
C12—C11—H11	120.0	C1—N1—S1	127.03 (19)
C13—C12—C11	120.7 (4)	O2—S1—O1	120.71 (14)
C13—C12—H12	119.6	O2—S1—N1	105.46 (12)
C11—C12—H12	119.6	O1—S1—N1	105.86 (13)
C14—C13—C12	120.4 (4)	O2—S1—C9	110.36 (14)
C14—C13—H13	119.8	O1—S1—C9	108.50 (14)
C12—C13—H13	119.8	N1—S1—C9	104.67 (13)
			
C6—C1—C2—C3	0.7 (4)	C6—C7—C15—C16	177.8 (2)
N1—C1—C2—C3	178.7 (3)	O3—C15—C16—C17	−37.3 (5)
C1—C2—C3—C4	0.5 (5)	C7—C15—C16—C17	140.8 (4)
C2—C3—C4—C5	−1.1 (5)	O3—C15—C16—C17A	106.8 (12)
C3—C4—C5—C6	0.4 (5)	C7—C15—C16—C17A	−75.1 (12)
C4—C5—C6—C1	0.8 (4)	O3—C15—C16—Br1A	−27.8 (5)
C4—C5—C6—C7	−179.8 (3)	C7—C15—C16—Br1A	150.3 (4)
C2—C1—C6—C5	−1.4 (4)	O3—C15—C16—Br1	87.4 (3)
N1—C1—C6—C5	−179.7 (2)	C7—C15—C16—Br1	−94.5 (3)
C2—C1—C6—C7	179.1 (3)	C7—C8—N1—C1	1.7 (3)
N1—C1—C6—C7	0.7 (3)	C7—C8—N1—S1	163.2 (2)
C5—C6—C7—C8	−179.2 (3)	C2—C1—N1—C8	−179.7 (3)
C1—C6—C7—C8	0.3 (3)	C6—C1—N1—C8	−1.5 (3)
C5—C6—C7—C15	0.7 (5)	C2—C1—N1—S1	20.1 (4)
C1—C6—C7—C15	−179.8 (3)	C6—C1—N1—S1	−161.7 (2)
C6—C7—C8—N1	−1.3 (3)	C8—N1—S1—O2	28.1 (2)
C15—C7—C8—N1	178.9 (3)	C1—N1—S1—O2	−174.2 (2)
C14—C9—C10—C11	0.7 (5)	C8—N1—S1—O1	157.1 (2)
S1—C9—C10—C11	−179.1 (3)	C1—N1—S1—O1	−45.2 (3)
C9—C10—C11—C12	0.0 (6)	C8—N1—S1—C9	−88.4 (2)
C10—C11—C12—C13	−0.8 (7)	C1—N1—S1—C9	69.3 (2)
C11—C12—C13—C14	1.1 (7)	C10—C9—S1—O2	−21.3 (3)
C12—C13—C14—C9	−0.4 (6)	C14—C9—S1—O2	158.9 (3)
C10—C9—C14—C13	−0.4 (5)	C10—C9—S1—O1	−155.7 (3)
S1—C9—C14—C13	179.3 (3)	C14—C9—S1—O1	24.6 (3)
C8—C7—C15—O3	175.7 (3)	C10—C9—S1—N1	91.7 (3)
C6—C7—C15—O3	−4.1 (5)	C14—C9—S1—N1	−88.0 (3)
C8—C7—C15—C16	−2.4 (4)		

### Hydrogen-bond geometry (Å, º)

*Cg*2 is the centroid of the C1–C6 ring.

**Table:**

*D*—H···*A*	*D*—H	H···*A*	*D*···*A*	*D*—H···*A*
C8—H8···O1^i^	0.93	2.57	3.312 (4)	137
C10—H10···O1^i^	0.93	2.44	3.280 (4)	150
C12—H12···*Cg*2^ii^	0.93	2.72	3.528 (2)	146

Symmetry codes: (i) −*x*+2, *y*+1/2, −*z*+1/2; (ii) *x*, −*y*+1/2, *z*−1/2.
